# First year medical students’ learning style preferences and their correlation with performance in different subjects within the medical course

**DOI:** 10.1186/s12909-017-0965-5

**Published:** 2017-08-08

**Authors:** Daniel Hernández-Torrano, Syed Ali, Chee-Kai Chan

**Affiliations:** 1 Nazarbayev University Graduate School of Education, Astana, Kazakhstan; 2 Department of Biomedical Sciences, School of Medicine, Nazarbayev University, Astana, Kazakhstan

**Keywords:** Learning styles, Academic performance, First-year, Medical education, Medical subject

## Abstract

**Background:**

Students commencing their medical training arrive with different educational backgrounds and a diverse range of learning experiences. Consequently, students would have developed preferred approaches to acquiring and processing information or learning style preferences. Understanding first-year students’ learning style preferences is important to success in learning. However, little is understood about how learning styles impact learning and performance across different subjects within the medical curriculum. Greater understanding of the relationship between students’ learning style preferences and academic performance in specific medical subjects would be valuable.

**Methods:**

This cross-sectional study examined the learning style preferences of first-year medical students and how they differ across gender. This research also analyzed the effect of learning styles on academic performance across different subjects within a medical education program in a Central Asian university. A total of 52 students (57.7% females) from two batches of first-year medical school completed the Index of Learning Styles Questionnaire, which measures four dimensions of learning styles: sensing-intuitive; visual-verbal; active-reflective; sequential-global.

**Results:**

First-year medical students reported preferences for visual (80.8%) and sequential (60.5%) learning styles, suggesting that these students preferred to learn through demonstrations and diagrams and in a linear and sequential way. Our results indicate that male medical students have higher preference for visual learning style over verbal, while females seemed to have a higher preference for sequential learning style over global. Significant associations were found between sensing-intuitive learning styles and performance in Genetics [β = −0.46, B = −0.44, *p* < 0.01] and Anatomy [β = −0.41, B = −0.61, *p* < 0.05] and between sequential-global styles and performance in Genetics [β = 0.36, B = 0.43, *p* < 0.05]. More specifically, sensing learners were more likely to perform better than intuitive learners in the two subjects and global learners were more likely to perform better than sequential learners in Genetics.

**Conclusion:**

This knowledge will be helpful to individual students to improve their performance in these subjects by adopting new sensing learning techniques. Instructors can also benefit by modifying and adapting more appropriate teaching approaches in these subjects. Future studies to validate this observation will be valuable.

## Background

Medical education and training have evolved and changed significantly in the last two decades. Medical students are expected to understand, retain and apply a challenging amount of knowledge and skills in a limited time during their training in medical school. To enable the students to learn effectively, progress have been made to improve teaching methods, moving away from traditional classroom-based didactic methods towards more student-centered, activity based strategies including problem-based learning, team-based learning and active learning.

Students commencing their medical training arrive with different educational and scholastic backgrounds and bring with them a diverse range of learning experiences that affect their success in medical education [1, 2]. As a consequence of these experiences the students invariably would have developed a preferred approach to acquiring and processing information. They would have adopted a learning strategy or style which they have found to be most effective for them as an individual shaped by their learning experiences and learning environment. In general terms, learning style is used to refer to “an individual's preferred way of gathering, organizing, and thinking about information” [3]. The term has enjoyed great popularity over the last decades based on the belief that knowing one’s preferred learning style, as well as the preferred approach to learning can be useful in informing the learner how they can adapt and improve to maximize their learning [4, 5]. Such knowledge of the class would also be critical to the instructor to ensure that an effective pedagogy employing a variety of teaching approaches, can best be developed and deployed to help the students to learn most effectively.

More than 70 learning styles models have been documented to date [6]. In the field of medical education, one of the most widely used is the Felder-Silverman model [7]. This model distinguishes four dimensions of learning styles: sensing-intuitive; visual-verbal; active-reflective; and sequential-global. According to the model students who prefer “sensing” are more concrete thinkers, adapt well towards facts, adept at memorizing while “intuitive” learners are abstract thinkers, innovative, loves patterns, concepts and relationships and oriented towards theories and underlying meanings. A “visual” learner prefers visual presentations in the form of charts, figures and diagrams while the “verbal” learner prefer written and spoken explanations. The “active” learner learns by trying things out, actively engaged and prefers working in a group. On the other hand, a “reflective” learner thinks things through and prefers working alone. Finally the “sequential” learner processes his or her thoughts in a linear fashion, building up stepwise using logical progression while a “global” learner thinks more holistically and is comfortable making big leaps, grasps the big picture and loves solving complex problems [8, 9]. This model has several advantages over other approaches, including the possibility of assessing multiple learning styles, brevity, ease of administration, free access and appropriate psychometric characteristics of the Index of Learning Styles (ILS). The instrument developed by the authors to measure learning styles is based on this model [10–12].

A considerable number of studies have analyzed the learning style profiles of medical students using the Felder-Silverman model. In general, research consistently evidences that the most common learning styles reported by medical students are sensing, visual, and sequential [12–15]. In addition, some studies have found that medical students have a predominantly reflective learning style preference [12, 13], although other studies have shown that medical students also exhibit an active learning style preference [15, 16]others found no preference for processing information actively or reflectively [12].

Studies analyzing gender differences in learning styles of medical students have yielded contradictory results. On the one hand, there is empirical evidence that gender has a significant influence on learning styles. For example, Hosford & Siders [12] found that a significantly higher number of female medical students preferred the sensing mode of learning as compared to males; whereas a significant number of male medical students preferred the visual mode. In addition, there seems to be research evidence that females prefer to learn by trying things (i.e., active learning style) rather than by thinking things through (i.e., reflective learning style) [15]. On the other hand, several studies have challenged the gender effect on learning preferences in medical students and found no evidence for the difference on learning styles between male and female medical students using the ILS instrument [16].

The relationship between learning styles and academic performance in medical education has been extensively studied but remains inconclusive. Some studies point out that there is no effect of learning styles on academic performance [15, 16]. On the other hand, other studies have demonstrated differences between the academic performance of students with different learning styles. For example, Hur & Kim [17] showed that Korean medical students with a reflective learning style preference performed significantly better in participation, problem solving, quiz, and team work. Furthermore, there is scientific evidence that students with intuitive learning preferences perform significantly better than students with sensing preferences in basic science, general pathology, and clinical pathology courses [18].

Research on students learning styles has shown that preferences of these learning styles also differ across different streams and courses from engineering to sciences, to humanities, architecture, pharmacy and health sciences [19–22]. However, little is understood about how learning styles impact learning and performance across different subjects within a course in medical education. There are a number of different subjects within a medical course. In the first year of their medical training most medical schools include in their curriculum basic biomedical subjects such as anatomy, cell pathology, genetics, immunology and skills in clinical research often named as evidence base medicine. The body of knowledge, end objectives of each of these courses, the teaching strategies and environment all play a part in shaping and reinforcing the learning strategy preferences of different groups of students. In this context, we aimed to examine the association of the different learning strategies with the students’ performance in different first-year medical subjects.

## Methods

Medical students who participated in the study were from a university in Astana, Kazakhstan. The students were from two cohorts of first year students from the Nazarbayev University School of Medicine. The participants of this study consist of 22 males and 30 females, their age ranges from 22 to 29 and they are all of Kazakhstan nationality except for one who is from Kyrgyzstan. The university is a western styled, research based university which uses English as medium for all teaching and learning and aims to generate and sustain excellence in health education and biomedical research and educate future medical leaders in health care, education and biomedical research.

Ethics approval for the study was obtained from the Institutional Research Ethics Committee (IREC) of Nazarbayev University, Astana, Kazakhstan. Informed consent was obtained from all participants of the study. At the beginning of the first year, medical students were given a letter of consent and information describing the project. Those who consented to participate were requested to complete the Index of Learning Styles Questionnaire obtained from North Carolina State University online site (http://www.engr.ncsu.edu/learningstyles/ilsweb.html). The questionnaire developed by Solomon and Felder consisted of 44 questions each with 2 options. This instrument which has been validated is designed to evaluate the students learning style preference on four dimensions based on the Felder-Silverman model [8, 9]. The results of the questionnaire were submitted online to obtain a learning style preference score for each individual showing across a range from negative 11 to positive 11 for four different categories, namely active-reflective, sensing-intuitive, visual-verbal and sequential-global. Students subsequently had access to their own scores and were informed of the implication of the scores for personal development and improvement. Their scores were collated and de-identified by a code. Results from the various subjects that the students took across the first year of the medical school were obtained and collated. A total of 52 students (42.3% males and 57.7% females) from two batches of first year medical school took part in the study. The students learning style preferences were analyzed and compared using educational research statistical software.

In the first year of the medical school, students take a number foundational Biomedical subjects in blocks lasting from two to 5 weeks which includes genetics, anatomy, immunology, microbiology, fuel metabolism, cell pathology, and a number of longitudinal course such as Ethics, Law and Professionalism, Evidence based medicine and Basic Physical Examination. Assessment of each subject is carried out using a combination of multiple choice questions which takes up about 60–70% of the subject marks and 30–40% is allocated to a performance score from active learning such as problem-based learning and team-based learning.

Correlations between the students’ learning styles across different subjects were analyzed using Pearson’s correlation and linear regression using SPSS and the Prism GraphPad software version 7.

## Results

### Students learning style distribution

**Fig. 1:**
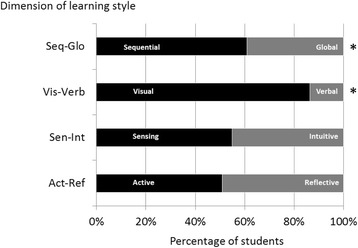
Learning style preferences of Year 1 Medical students. Each bar indicates the percentage of the cohort’s preference for each category within a dimension. * indicate the two dimensions with difference in preference for a particular learning style of more than half

**Fig. 2:**
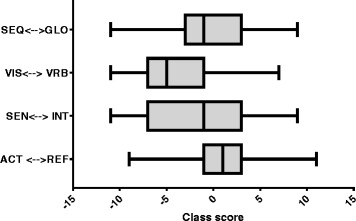
Box whisker plot showing preferential learning styles of 1st year medical students indicating range, upper quartile (25th percentile), median and lower quartile (lower 25th percentile). Class score was obtained by analyzing the student’s score for each of the 4 dimensions of sequential-global, visual-verbal, sensing-intuitive and active-reflective. The maximum score for each category is +11 or −11

Student learning styles preferences of two batches of first year medical students were analyzed across four dimensions of learning styles namely sequential-global, verbal-visual, intuitive-sensing and reflective-active. The analysis detected very little difference in the number of students preferring sensing (54.9%) as compared to those who prefer the intuitive (45.1%) in their learning styles, the same was uncovered with regards to those preferring reflective (49.1%) versus those who prefer the active (50.9%) learning styles (see Fig. 1). This is not however to say that there are no student or very few that who are very clearly sensing in their approach in learning, as seen in Fig. 2, in which the median for sensing-intuitive is −0.9 but the spread is substantial with the lower quartile at −6.5 and the upper quartile at 3.3. The median for active-reflective is 0.7 and the upper and lower quartiles are clearly more narrow, nevertheless there are those in class that are clearly very active and those that are very reflective as shown by the upper and lower limit of the plot.

When comparing global-sequential preference however, more of the first year medical students were sequential in their learning style (60.5%) as compared to global (39.5%) as seen in Fig. 1. But very clearly though there is a great difference in the preference of the students to visual (80.8%) compared to verbal (19.2%). The median for sequential-global is −0.8 and the quartiles were −2.7 and +3.0 (Fig. 2) while the median for visual-verbal was −5.1 and the quartiles were −6.8 and −0.9.

### Learning style preference by gender

**Fig. 3:**
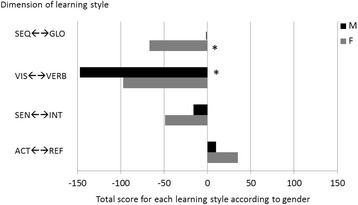
Learning style preferences by gender. For each dimension of learning style, the scores of the students for each gender were added to give a final sum. The score indicate the cumulative strength of the gender specific preference rather than the mean. * indicate the two dimensions with the biggest difference for learning styles between male and female

When the scores of the students for each category of learning style preference were added up for each of the two axes and compared across gender (Fig. 3), there was a clear difference for sequential preference versus global. The cumulative score for males was 0 while the score for females was −69 indicating a preference for sequential for females while the score for male indicated an equal preference for sequential versus global. The clear preference for visual learning style by both males and females was even more noticeable with a score of −147 for males and a lower score for female of −97. While both males (−16) and females (−49) preferred the sensing learning style as compared to intuitive, the difference between gender was not so significant. The same was detected for the preference of reflective learning versus active for both females (35) and males (10), the difference between the male and the females was not so substantial.

### Learning styles preference and performance in medical subjects

We were interested to investigate if the preference of a particular learning style would be associated with the increase in the performance score of certain subjects in the first year medical course. The performance score for first year Anatomy and Genetics were analyzed and linear regression and a Pearson correlation analysis was carried out for the two subjects for each of the learning styles.

The linear regression analysis for Genetics is as shown in Table 1. The linear regression for Genetics and the various learning styles showed no significant association for active-reflective, and visual-verbal learning style preference but was significant for the sensing-intuitive learning style preference with β value of −0.46 and *B* value of −0.44 and 95% CI of −0.73 to −0.14 and *p* value <0.01 and for sequential-global with β value of 0.36 and *B* value of 0.43 and 95% CI of 0.07 to 0.97 and *p* value <0.05 (Table 1).

**Table 1:** Predictive ability of learning styles preferences over academic performance on genetics and anatomy subjects

	Zero-order correlation						Genetics			Anatomy		
Variable	1	2	3	4	5	6	β	*B*	*95% CI*	β	*B*	*95% CI*
1. Act. – Ref.	-	−.30^*^	.36^**^	−.10	.21	.08	.16	.20	[−0.16, −0.57]	−.06	−.12	[−0.73, 0.48]
2. Sen. – Int.		-	−.21	.47^***^	.31^*^	−.39^**^	−.46^**^	−.44	[−0.73, −0.14]	−.41*	−.61	[−1.09, −0.13]
3. Vis. – Verb.			-	.14	−.03	.14	−.14	.16	[−0.49, 0.17]	.08	.15	[−0.39, 0.69]
4. Seq. – Glo.				-	.14	−.15	.36^*^	.43	[0.07, 0.97]	.05	.09	[−0.49, 0.69]
5. Genetics					-	.47^***^						
6. Anatomy						-	*F* (4, 50) = 3.47, *p* = .015, = *R* ^*2*^ = .23			*F* (4, 49) = 2.10, *p* = .096, *R* ^*2*^ = .16		

Note. *Act* Active, *Ref* Reflective, *Sen* Sensing, *Int* Intuitive, *Vis* Visual, *Verb* Verbal, *Seq* Sequential, *Glo* Global

*p < .05, **p < .01, ***p < .001

When the linear regression and Pearson’s correlation was calculated for Anatomy, there was also a similar significant association between performance in Anatomy and sensing-intuitive with β value of −0.41 and *B* value of −0.61 and 95% CI of −1.09 to −0.13 and *p* value <0.05 (see Table 1). There were however no associations with the other learning style preferences.

## Discussion

This study is a first that analyses the learning style preferences, gender differences, and the association of learning style preferences with the performance of individual medical subjects in a medical course in the context of a Central Asian medical school. The students were from the first-year medical course and had a variety of subjects ranging from Anatomy, Immunology, Genetics, Microbiology, Cell Pathology, Evidence based Medicine, Introductory Physical Examination and Ethics. Although previous studies have shown association of learning preferences with the over-all performance in the medical course such as GPA, we show for the first time the learning style preference with specific subjects within the medical course.

In general, our results indicated that students show preferences for visual and sequential learning styles. This suggests that medical students in our sample preferred to learn through demonstrations, photographs, and diagrams and in a linear and sequential way, as it has been evidenced elsewhere ^8–11^. However, no significant differences were found among their preferences in the continuous sensing-intuitive and active-reflective.

As been shown by previous studies [12, 15] we also found differences in learning styles based on gender. Our results indicated that male medical students have higher preference for visual learning style over verbal compared to females, although it was also clear that both males and females from the first-year medical school preferred visual learning style as compared to verbal. It is interesting to note that although there was a clear preference for visual learning style over verbal there was no correlation between performance in Anatomy or Genetics with the preference for visual leaning style. The students in our study may have preferred to learn and acquire knowledge through visual means but that did not seem to impact their performance, at least not in Anatomy and Genetics. Females seemed to also have a higher preference for sequential learning style over global as compared to males. This seems to complement the findings of Hosford and Siders^16^ that females more likely than males learn less by global jumps and more by stepwise understanding and logical progression.

Performance in Anatomy and Genetics was analyzed and significant associations were found for performance in the two subjects with the sensing learning style over the intuitive learning style. In addition performance in Genetics also has a significant association with the global learning style while no associations were found for the other learning styles preferences.

The association of performance in Genetics with sensing learning style over intuitive is not too surprising as there is a need to remember significant quantity of specific facts in Genetics although conceptual learning is still critical in the mastery of the subject. Sensing learners are thought to be better at learning facts and memorization and enjoy more hands-on laboratory activities, while intuitive learners appreciate learning through connections, relationships, concepts and patterns. This was also supported by the same finding in Anatomy with a lower significant *p* value in which performance for the subject was less strongly associated with preference for sensing learning style over intuitive, as demonstrated by smaller β and *B* value. Interestingly performance in Genetics is strongly associated with those having preference for the global learning style, and we hypothesize that this is an indication that those who are better able to integrate their Genetics knowledge in the bigger picture of medicine especially in clinical applications, are able to perform better in the subject.

Knowledge of the learning style preference for an individual student is helpful, since with this knowledge the student is better able to understand himself or herself, and is better able to exploit to this knowledge to his or her advantage to maximize learning. Uncovering the association of performance of individual subjects to specific learning styles will help students to examine his or her own need to expand the ways in which the student chooses to learn, and train to learning differently in order to perform better. A student who is predominantly learning through intuitive ways and struggles in subjects like anatomy and genetics will have to learn and practice to acquire knowledge using sensing methods in order to perform better. They can benefit to learn using sensing learners’ strategies to memorize specific details and facts through the use of acrostics and memory cards for example. Pedagogically it is also valuable to the instructor and teacher, not just to know the learning style preference of the class and the individuals but also to teach effectively in specific subjects, and to have activities that enables more of “sensing” learning, such as providing drills and practices for factual memorization in Genetics and Anatomy, although there is a need not to neglect the conceptual understanding of principles in Genetics and Anatomy. The instructors can also emphasize the need to integrate Genetics knowledge into useful clinical applications using clear examples. It will be useful and interesting to investigate and to validate this strategy in future studies.

### Limitations and future research

This is an initial study to look at the association of learning style preferences with performance in individual subjects in a first-year medical course, we have only examined two subjects from a range of subjects in the course and the cohort size was relatively small which is one of the limitations of this study, moreover the participants are all Kazakhstanis except for one who is from Kyrgyzstan. As such the findings may not be truly representative of all medical students in different medical schools in diverse geographical locations and teaching environments across the world and further studies in the future to validate this observation will be valuable. In addition, participants were selected using non-probabilistic sampling procedures, which limits the generalization of the findings. Although we have demonstrated significant associations it will be useful to continue with future studies, to include more samples and analyzing across more subjects. It will be also fascinating to carry out a longitudinal study to see how learning style preference may change as the medical student progresses and how it impacts his or her performance in individual subjects. Finally, this study did not control the effect of variables such as intelligence or personality, which have consistently demonstrated predictive capacity over academic performance in the literature. Future studies should consider the mediating effect that these variables might have on the relationship between learning styles and academic performance.

## Conclusion

We have shown early evidence that different student learning style preference is associated with performance in specific subjects in a first-year medical course and that sensing learning style is associated with performance in Genetics and Anatomy. We postulate that this knowledge will be useful in helping the individual student to improve their performance in these subjects by adopting new sensing and global learning techniques to maximize learning. This finding will also be helpful to teachers and instructors as they can benefit by modifying and adapting more appropriate teaching approaches in these subjects; however future studies to validate this observation will be valuable.

## Abbreviations

Act-Ref: Active-Reflective
Sen-Int: Sensing-Intuitive
Seq-Glo: Sequential-Global
Vis-Verb: Visual-Verbal
